# Association of IL-10 gene promoter polymorphisms with susceptibility to pseudoexfoliation syndrome, pseudoexfoliative and primary open-angle glaucoma

**DOI:** 10.1186/s12881-020-0969-6

**Published:** 2020-02-12

**Authors:** Ghasem Fakhraie, Farshid Parvini, Jalaledin Ghanavi, Shima Saif, Poopak Farnia

**Affiliations:** 1 Department of Ophthalmology, Glaucoma Service, Farabi Eye Hospital, Tehran University of Medical Sciences, Tehran, Iran; 2 Department of Biology, Faculty of Basic Sciences, Semnan University, Semnan, Iran; 3 Mycobacteriology Research Centre (MRC), National Research Institute of Tuberculosis and Lung Disease (NRITLD), Shahid Beheshti University of Medical Sciences, Tehran, Iran; 4 Department of Biotechnology, School of Advanced Technologies in Medicine, Shahid Beheshti University of Medical Sciences, Tehran, Iran

**Keywords:** IL-10 gene, Polymorphism, PEX, PEXG, POAG

## Abstract

**Background:**

The involvement of cytokines in pathogenesis of pseudoexfoliation syndrome and glaucoma has been demonstrated in several studies. The aim of the present study was to explore the association between three promoter polymorphisms −592C/A (rs1800872), − 819C/T (rs1800871) and -1082A/G (rs1800896) of interleukin 10 (IL-10) gene with susceptibility to pseudoexfoliation syndrome (PEX), pseudoexfoliative glaucoma (PEXG), and primary open-angle glaucoma (POAG).

**Methods:**

In this study, 114 PEX, 118 PEXG, 114 POAG patients and 126 healthy individuals from Iranian population were participated. Detailed ophthalmic examinations by an ophthalmologist including slit-lamp bio-microscopic examination, dilated examination of the lens, gonioscopy, and funduscopy were carried out on patients and controls. Genomic DNA was extracted from the blood samples and ARMS–PCR was performed to detect promoter polymorphisms of IL-10.

**Results:**

In all three SNPs studied, there was a significant difference in the genotype distribution between patients and control subjects. Results revealed that the AA genotype of IL-10 -592C/A SNP is associated with PEX. However, TT genotype of −819C/T and AA genotype of -1082A/G SNP are significantly associated with susceptibility to either PEX or PEXG and POAG disorders. Furthermore, the ACC haplotype containing the IL-10 -1082A allele was associated with PEX (*P* = 0.02, OR = 5.76, 95% CI = 5.17–24.49), PEXG (*P* = 0.006, OR = 7.54, 95% CI = 6.62–30.76) and POAG (*P* = 0.003, OR = 8.11, 95% CI = 7.13–33.15).

**Conclusions:**

Our results demonstrated that IL-10 gene promoter polymorphisms are associated with susceptibility to PEX, PEXG and POAG in Iranian population. Considering the fact that IL-10 polymorphisms are associated with various IL-10 expressions, further research is needed to explain its involvement in these disorders and the formation of extracellular fibrillar amyloid deposits in PEX and PEXG.

## Background

Pseudoexfoliation (PEX) syndrome, as an age-related disorder of the extracellular matrix is characterized by pathologic accumulation of abnormal fibrillar material in various intra- and extraocular tissues [1]. PEX syndrome is the most common cause of secondary glaucoma, named pseudoexfoliative glaucoma (PEXG) which is considered by higher intraocular pressures (IOP), more serious clinical course, more rapid progression, and worse prognosis compared to primary open-angle glaucoma (POAG) [2].

Although the exact etiology and pathogenesis of PEX, PEXG and POAG disorders are still not known, the strong pattern of familial aggregation [3] as well as genome-wide [4] and case-control [5–7] association studies indicate significant genetic contribution to pathology of these disorders. Furthermore, the association of microRNA-related polymorphisms with PEX, PEXG and POAG disorders have been recently investigated [8]. Therefore, identification of the genes causing or modifying PEX/PEXG/POAG phenotype remains a challenge for researchers working on pathology and pathophysiology of these three eye disorders. Such genetic data may not only provide a better understanding of the molecular and pathogenesis mechanisms underlying PEX, PEXG and POAG disorders, but also enable the possible development of new drugs or treatments.

Previous studies have shown the expression relationship of cytokines with pseudoexfoliation syndrome and glaucoma pathology [9–11]. Interleukin-10 (IL-10) is a potent anti-inflammatory cytokine which plays a central role in anti-inflammation [12]. Three common single nucleotide polymorphisms (SNPs) -592C/A (rs1800872), −819C/T (rs1800871) and -1082A/G (rs1800896) have been previously reported in the promoter region of IL-10 gene. These SNPs control the transcription of IL-10 mRNA and IL-10 protein expression in vitro [13]. To date, several studies have exhibited associations between -592C/A, −819C/T and -1082A/G promoter SNPs with susceptibility to aging [14], risk of cancer [15–17], schizophrenia [18], Alzheimer’s disease [19–23], acute myocardial infarction (AMI) [24] and inflammatory bowel disease (IBD) [25]. Researchers have indicated that IL-10 -592C/A and -819C/T SNPs can be predictive factors for the pathogenesis of POAG in the Chinese population [26]. However, no association studies have been conducted in order to investigate the possible relationships between -592C/A, −819C/T and -1082A/G promoter polymorphisms and susceptibility to all three PEX, PEXG and POAG disorders.

The aim of the present study was to explore the possible association of three IL-10 gene promoter SNPs -592C/A (rs1800872), −819C/T (rs1800871) and -1082A/G (rs1800896), and their related haplotypes with susceptibility to PEX, PEXG and POAG eye disorders in well-characterized patient groups originating from Iran. To best of our knowledge, this study is the first comprehensive report on these polymorphisms in PEX, PEXG and POAG patients from Iranian population and other populations worldwide.

## Methods

### Subjects and diagnostic criteria

A total of 346 unrelated patients including 114 PEX, 118 PEXG and 114 POAG cases, as well as 126 healthy controls were recruited for study participation. Patients and healthy controls were all of Iranian origin. This research was a collaborative work between Glaucoma Service of Farabi Eye Hospital and Shahid Beheshti University of Medical Sciences. The study protocol was approved by the Ethical Committees of Shahid Beheshti University of Medical Sciences (IR.SMBU.NRITLD.REC.1395.254). The research was performed according to the tenets of the Declaration of Helsinki for research involving human subjects.

### Clinical study protocol

Prior to clinical examination, a questionnaire was administered including demographic and ophthalmologic data as well as medical history [27]. Then, all subjects underwent detailed ophthalmic examinations by an ophthalmologist including slit-lamp bio-microscopic examination, dilated examination of the lens, gonioscopy, and funduscopy.

Subjects with PEX were defined as those with clinical evidences of pseudoexfoliation on the anterior capsule, and pupillary margin in mydriasis. Prior to pupil dilation, a detailed high-magnification slit-lamp assessment of the pupil margin was performed. Then, the anterior lens surface from each eye was examined using a narrow slit-lamp beam to scan the lens left to right, followed by examination using a vertical broad slit-lamp beam, looking specifically for early signs of PEX. The other main predictor was an IOP of less than 21 mmHg as well as no clinical evidence of glaucomatous optic neuropathy. All PEXG patients were defined as those with clinical evidences of PEX in either eye, elevated IOP, and glaucomatous optic neuropathy (defined as loss of neuroretinal rim with a vertical cup-to-disc ratio greater than 0.7) with compatible visual field loss. The diagnostic criteria for POAG were applied including exclusion of congenital glaucoma, exfoliation syndrome, or other secondary causes of glaucoma in either eye; gonioscopically open anterior chamber angle (Shaffer grade III or IV); characteristic optic disc changes; characteristic visual field changes according to Anderson’s criteria [28], and IOP greater than 22 mmHg in both eyes without medications. With respect to control subjects, they all had IOP below 20 mmHg, and no glaucomatous disc damage, no PEX material deposits on anterior lens capsule and/or pupillary margin, no criteria indicating early or suspect PEX and no family history of PEX and glaucoma.

### DNA extraction and genotyping

Genomic DNAs were extracted from peripheral blood leukocytes of 114 PEX, 118 PEXG and 114 POAG affected patients and 126 healthy controls using the standard phenol-chloroform procedure, with slight modifications [29]. Amplification Refractory Mutation System-Polymerase Chain Reaction (ARMS-PCR) was performed for genotyping of -592C/A, −819C/T and -1082A/G SNPs using primer pairs (Additional File 1: Table S1) as described by Abanmi and colleagues [30] with slight modifications. Human growth factor hormone primers (Additional File 1: Table S1) were included in each PCR reaction as internal control. Briefly, genomic DNA was amplified with the use of Taq DNA polymerase in two different PCR reactions for each polymorphism; each reaction employed a generic antisense primer and one of the two allele specific sense primers. In order to assess the success of PCR amplification in both reactions, 429 bp of Human growth factor hormone gene (accession number M13438) was amplified as an internal control. PCR conditions are summarized in Additional File 1: Table S2.

## Statistical analysis

Allele, genotype and haplotype frequencies were compared between patients and control groups using chi-squared test (× 2) or Fisher’s exact test where appropriate. In order to evaluate the association between the specific alleles, genotypes and IL-10 promoter haplotypes with susceptibility to PEX, PEXG and POAG disorders in patients compared to controls, chi-square and odds ratios (ORs) were estimated within 95% confidence intervals. A *p*-values of less than 0.05 were considered statistically significant. Additionally, the association between haplotypes of IL-10 gene -592C/A, −819C/T and -1082A/G polymorphisms and risk of PEX/PEXG/POAG was assessed by multiple logistic regression analysis. All statistical analysis was performed using SPSS Software, version 22 (SPSS Inc., Chicago, Illinois, USA).

## Results

### Genotype and allele frequencies of IL-10 gene promoter polymorphisms

In this study, according to the results of clinical examination performed by the ophthalmologist on patients and controls, 114 PEX, 118 PEXG, 114 POAG and 126 healthy subjects were selected. The genotype and allele frequencies of IL-10 gene promoter SNPs -592C/A, −819C/T and -1082A/G in control subjects and PEX, PEXG and POAG affected patients are shown in Table 1. With respect to genotype frequencies (AA vs. AC and CC), at position -592C/A, significant difference was observed for the frequency of AA genotype in PEX patients (but not in PEXG and POAG patients) compared to controls (14% vs. 3.2%). At position -819C/T (TT vs. TC and CC), frequencies of TT genotype (14, 8.5 and 10.5% for PEX, PEXG and POAG patients, respectively vs. 1.6% in controls) demonstrated significant differences between patients and healthy controls. Similar results were obtained for -1082A/G polymorphism, so that comparison of AA genotype frequency in PEX, PEXG and POAG patients (19.3, 20.3 and 21.3%, respectively) exhibited a significant difference compared to controls (1.6%) (Table 1). Totally, among the studied genotypes, TT and AA genotypes of -819C/T and -1082A/G SNPs, respectively, were much more frequent in PEX, PEXG and POAG patients in comparison with healthy controls. However, at position -592C/A, higher frequency of AA genotype was observed in just PEX affected patients compared to controls.

**Table 1:** Distribution of alleles and genotypes of *IL-10* SNPs

		Alleles			Genotypes			Recessive *p-*value	
-592 (rs1800872)	*n*	A	C	*p-*Value	AA	AC	CC	AA vs. AC + CC	OR (95% CI)
PEX, *n* (%)	114	130 (57.02)	98 (42.98)	0.39	16 (14)	98 (86)	0 (0)	0.04	4.97 (4.45–21.08)
PEXG, *n* (%)	118	128 (54.24)	108 (45.76)	0.68	10 (8.5)	108 (91.5)	0 (0)	0.26	NA
POAG, *n* (%)	114	126 (55.26)	102 (44.74)	0.57	12 (10.5)	102 (89.5)	0 (0)	0.14	NA
Control, *n* (%)	126	130 (51.59)	122 (48.41)	Ref group	4 (3.2)	122 (96.8)	0 (0)	Ref group	–
-819 (rs1800871)	*n*	T	C	*p-*Value	TT	TC	CC	TT vs. TC + CC	OR (95% CI)
PEX, *n* (%)	114	130 (57.02)	98 (42.98)	0.39	16 (14)	98 (86)	0 (0)	0.004	10.12 (13.29–71.61)
PEXG, *n* (%)	118	128 (54.24)	108 (45.76)	0.68	10 (8.5)	108 (91.5)	0 (0)	0.03	5.74 (7.98–43.34)
POAG, *n* (%)	114	126 (55.26)	102 (44.74)	0.57	12 (10.5)	102 (89.5)	0 (0)	0.01	7.29 (9.89–53.53)
Control, *n* (%)	126	126 (50)	126 (50)	Ref group	2 (1.6)	122 (96.8)	2 (1.6)	Ref group	–
-1082 (rs1800896)	*n*	A	G	*p-*Value	AA	AG	GG	AA vs. AG + GG	OR (95% CI)
PEX, *n* (%)	114	132 (57.89)	96 (42.11)	0.22	22 (19.3)	88 (77.2)	4 (3.5)	0.02	7.29 (6.37–29.69)
PEXG, *n* (%)	118	130 (55.08)	106 (44.92)	0.43	24 (20.3)	82 (69.5)	12 (10.2)	0.002	7.79 (6.76–31.39)
POAG, *n* (%)	114	130 (57.02)	98 (42.98)	0.28	24 (21.1)	82 (71.9)	8 (7)	0.005	8.13 (7.06–32.82)
Control, *n* (%)	126	126 (50)	126 (50)	Ref group	4 (3.2)	118 (93.6)	4 (3.2)	Ref group	–

### Haplotype analysis of IL-10 gene promoter polymorphisms

Based on the obtained results, five different haplotypes including GCC (reference haplotype), ACC, GTA, ATA and GCA were investigated in studied population. Constituents of the haplotypes were written in the order of -1082A/G, −819C/T and -592C/A of the IL-10 gene promoter. The frequencies of these haplotypes for PEX, PEXG and POAG affected patients and control subjects are summarized in Table 2. According to this table, the frequency of ACC haplotype in PEX (14%), PEXG (18.6%) and POAG (19.3%) patients are much higher than healthy controls (3.2%). However, no significant differences were observed between the other studied haplotypes in both groups.

**Table 2:** Haplotype structures and frequencies in the IL-10 gene promoter

Subjects	N	GCC	ACC	GTA	ATA	GCA
PEX, n (%)	114	82 (71.9)^a^	16 (14)	10 (8.8)	6 (5.3)	0 (0)
Control, n (%)	126	118 (93.6)	4 (3.2)	2 (1.6)	0 (0)	2 (1.6)
*p*-value		Ref. group	0.02	0.08	0.07	0.51
OR (95% CI)		–	5.76 (5.17–24.49)	NA	NA	NA
PEXG, n (%)	118	86 (72.9)	22 (18.6)	8 (6.8)	2 (1.7)	0 (0)
Control, n (%)	126	118 (93.7)	4 (3.2)	2 (1.6)	0 (0)	2 (1.6)
*p*-value		Ref. group	0.006	0.16	0.42	0.51
OR (95% CI)		–	7.54 (6.62–30.76)	NA	NA	NA
POAG, n (%)	114	80 (70.2)	22 (19.3)	10 (8.8)	2 (1.8)	0 (0)
Control, n (%)	126	118 (93.7)	4 (3.2)	2 (1.6)	0 (0)	2 (1.6)
*p*-value		Ref. group	0.003	0.08	0.41	0.51
OR (95% CI)		–	8.11 (7.13–33.15)	NA	NA	NA

^a^ Numbers of haplotypes are shown with their frequencies in parentheses. Constituents of the haplotypes are written in the order of −1082 G/A, −819 C/T and – 592 C/A SNPs of *IL-10*

### Association of studied polymorphisms/haplotypes with susceptibility to PEX, PEXG and POAG eye disorders

Associations between three studied promoter polymorphisms of IL-10 gene with susceptibility to PEX, PEXG and POAG eye disorders were evaluated. With regard to -592C/A polymorphism, the AA genotype significantly increased the susceptibility to PEX disorder (*P* = 0.04, OR = 4.97, 95% CI = 4.45–21.08) compared to the wild-type genotypes. However, no association was found between this genotype and susceptibility to PEXG and POAG disorders (Table 1). In regards to -819C/T promoter position, significant association was observed between TT genotype with susceptibility to PEX (*P* = 0.004, OR = 10.12, 95% CI = 13.29–71.61), PEXG (*P* = 0.03, OR = 5.74, 95% CI = 7.98–43.34) and POAG (*P* = 0.01, OR = 7.29, 95% CI = 9.89–53.53). Similarly, the AA genotype of -1082A/G promoter SNP was significantly associated with susceptibility to PEX (*P* = 0.02, OR = 7.29, 95% CI = 6.37–29.69), PEXG (*P* = 0.002, OR = 7.79, 95% CI = 6.76–31.39) and POAG (*P* = 0.005, OR = 8.13, 95% CI = 7.06–32.82) (Table 1).

At the haplotype level, the ACC haplotype was significantly associated with susceptibility to PEX (*P* = 0.02, OR = 5.76, 95% CI = 5.17–24.49), PEXG (*P* = 0.006, OR = 7.54, 95% CI = 6.62–30.76) and POAG (*P* = 0.003, OR = 8.11, 95% CI = 7.13–33.15). However, no statistically significant association was found between ATG, ATA and ACG haplotypes with PEX, PEXG and POAG (Table 2). Using logistic multivariate regression analysis, association of ACC haplotype with susceptibility to PEX (*P* = 0.002, OR = 5.76, 95% CI = 1.86–17.84), PEXG (*P* = 0.001, OR = 7.55, 95% CI = 2.51–22.69) and POAG (*P* = 0.001, OR = 8.11, 95% CI = 2.69–24.43) was observed. Similar association was obtained between GTA haplotype and susceptibility to PEX (*P* = 0.012, OR = 7.19, 95% CI = 1.53–33.70), PEXG (*P* = 0.034, OR = 5.49, 95% CI = 1.14–26.49) and POAG (*P* = 0.011, OR = 7.37, 95% CI = 1.57–34.56). However, no statistically significant association was found between ATA and ACG haplotypes and the risk of PEX, PEXG and POAG disorders (Table 3).

**Table 3:** Logistic multivariate regression analysis of the association between IL-10 promoter polymorphisms and risk of PEX/PEXG/POAG disorders

Disorder/Haplotype	*β*	S.E.	Wald	*P*	OR (95% CI)
PEX					
GCC					Ref. group
ACC	1.75	.58	9.19	.002	5.76 (1.86–17.84)
GTA	1.973	.79	6.27	.012	7.19 (1.53–33.70)
ATA	21.57	1.6E+ 4	.01	.999	NA
GCA	−20.84	2.8E+ 4	.01	.999	NA
PEXG					
GCC					Ref. group
ACC	2.021	.56	12.94	.001	7.55 (2.51–22.69)
GTA	1.703	.80	4.49	.034	5.49 (1.14–26.49)
ATA	21.52	2.8E+ 4	.01	.999	NA
GCA	−20.89	2.8E+ 4	.01	.999	NA
POAG					
GCC					Ref. group
ACC	2.09	.56	13.85	.001	8.11 (2.69–24.43)
GTA	1.99	.79	6.43	.011	7.37 (1.57–34.56)
ATA	21.59	2.8E+ 4	.01	.999	NA
GCA	−20.81	2.8E+ 4	.01	.999	NA

## Discussion

Several studies have previously explored the role of different genes or loci in PEX, PEXG, and POAG eye disorders [4, 5, 7, 8, 31]. These findings have indicated that the genetic mechanisms involved in pathogenesis of these disorders are complex. Therefore, the existence of additional susceptibility loci for PEX, PEXG, and POAG remains to be identified.

It has been shown that pro-inflammatory cytokines are involved in the pathology and pathophysiology of pseudoexfoliation syndrome/glaucoma [11, 32]. Interleukin-10 (IL-10) is known to play key roles in immune-regulating and anti-inflammatory responses. The present study was conducted in order to investigate the possible association of three promoter SNPs -592C/A, −819C/T and -1082A/G of IL-10 gene as well as their related haplotypes with susceptibility to PEX, PEXG, and POAG in Iranian population. AA genotype of -592C/A SNP significantly increased the susceptibility to PEX disorder compared to controls. However, the AA genotype of -1082A/G SNP, showed a strong association with susceptibility to either PEX (OR = 7.29) or PEXG (OR = 7.79) and POAG (OR = 8.13). In regards to -819C/T SNP, much higher frequency of TT genotype in studied patients compared to control group clearly suggested a significant association of this genotype with susceptibility to PEX, PEXG and POAG. It has been reported that individuals carrying either AA genotype of -592C/A or CC genotype of -819C/T are associated with an increased risk of POAG in Chinese population [26]. Several researchers have found similar associations between -592AA, −819TT and -1082AA genotypes with susceptibility to different diseases. Based on experimental evidences, it has been revealed that subjects with rheumatoid arthritis show higher frequency of -592AA [33, 34]. Also, patients suffering from asthma demonstrated -592AA and -1082AA genotypes more frequently [35], while -592AA and -819TT genotypes were more observed in prostate and colon cancers [36, 37]. A recent study has indicated that -819TT and AA prevalence is significantly higher in AML cases compared to the healthy controls [24]. Moreover, it has been found that the frequency of IL-10 -819 T/T is apparently increased in higher age groups and it is suggested that IL-10 -592A/C and -819 T/C can be an appropriate candidate as an aging-related gene [14]. Based on the findings of several researches, it seems that Alzheimer’s disease and glaucoma have some features in common since they are both age-related neurodegenerative diseases. Results of the present study revealed higher prevalence of AA genotype of -1082A/G SNP in studied eye disorders. Similarly, a significant increase of the -1082A allele has been reported in Alzheimer’s disease cases compared to controls [19]. Also, meta-analysis has recently revealed an association between IL-10 -1082A/G and the risk of developing the Alzheimer’s disease in a Brazilian cohort, in a way that A allele carriers (AA+AG) demonstrated a higher risk of the disease in comparison with the homozygote GG [23]. Moreover, in present work the association of IL-10 promoter haplotypes with susceptibility to PEX, PEXG, and POAG was evaluated which demonstrated an association of the ACC haplotype containing the IL-10 -1082A allele with PEX, PEXG and POAG. This finding revealed a very little linkage disequilibrium between IL-10 -1082 position with -592C/A and -819C/T SNPs, suggesting that the effect is largely attributable to IL-10 -1082A/G polymorphism. While we assessed the association of IL-10 promoter haplotypes and risk of PEX/PEXG/POAG by multivariate regression analysis, association of ACC and GTA haplotypes with susceptibility to all three studied disorders was observed. However, despite the absence of the association of ATA and GCA haplotypes with the studied eye disorders, the lack of ATA haplotype as well as very low frequency of GTA and GCA haplotypes in control group compared to patients clearly validated the reliability of the association found between AA, TT and AA genotypes of -592C/A, −819C/T and -1082A/G polymorphisms, respectively, with susceptibility to PEX, PEXG and POAG in the studied population. Previous studies have found that the ACA (OR = 2.60, 95% CI, 1.48–4.58) and GTA (OR = 2.34, 95%CI, 1.42–3.86) haplotypes were associated with an increased risk of POAG, while the ATC haplotype showed a decreased risk of POAG (OR = 0.63, 95% CI, 0.49–0.81) [26]. This result might be due to different ethnicity and eventually smaller sample size of the present study. Other studies have reported similar associations between IL-10 -592C/A, − 819C/T and -1082A/G haplotypes and susceptibility to diseases. It has been shown that GCC/ACC haplotype is more frequent in Alzheimer’s disease cases compared to controls [22]. Moreover, the haplotype -1082A/− 819 T is shown to be associated with a higher risk of developing the Alzheimer’s disease [20]. On the other hand, the IL-10 promoter SNPs may affect IL-10 mRNA and protein expression [13, 38]. As an instance, it has been confirmed by reverse transcriptase-PCR that -819TT/−592AA haplotype was a determinant of high IL-10 transcription and mRNA transcription in lipopolysaccharide-stimulated peripheral blood mononuclear cells [39]. New findings have demonstrated that serum level of the cytokine IL-10 is increased in patients with Alzheimer’s disease compared to vascular dementia and Down syndrome and healthy controls [40]. From molecular point of view, over-expression of IL-10 exacerbates the Alzheimer’s disease in mouse model by unexpectedly giving rise to deposition of amyloid-β (Aβ) plaques in brain [41]. In a recent research work, the IL-10 deficiency induced by knocking out the IL-10 gene in Alzheimer’s-afflicted mice model is shown to promote the clearance of Aβ plaques from the brain and improves memory restoration [42]. Surprisingly, PEX and PEXG are characterized by ocular accumulation of fibrils containing amyloid-related proteins [43]. As demonstrated by extensive evidences, extracellular fibrillar amyloid deposits are accumulated in retinal ganglion cells, the walls of iris arterioles, lens capsule and occasionally cornea of PEX and PEXG patients [44–46]. Based on these findings, it might be suggested to assess whether the IL-10 polymorphisms can be influential on fibrillar amyloid deposition in PEX and PEXG glaucoma.

The findings of the present study can be evaluated on a larger sample size. In addition, it is recommended to perform the assessment among other racial groups. Furthermore, it seems interesting to evaluate the PEX, PEXG and POAG patients having -592C/A, −819C/T and -1082A/G polymorphisms for the incidence of Alzheimer’s disease.

## Conclusions

In conclusion, our results revealed that while the AA genotype of IL-10 -592C/A SNP is just associated with PEX, the AA and TT genotypes of IL-10 promoter -1082A/G and -819C/T polymorphisms, respectively, are associated with susceptibility to PEX, PEXG and POAG in Iranian population. ACC haplotype containing the IL-10 -1082A allele was associated with PEX (*P* = 0.02, OR = 5.76, 95% CI = 5.17–24.49), PEXG (*P* = 0.006, OR = 7.54, 95% CI = 6.62–30.76) and POAG (*P* = 0.003, OR = 8.11, 95% CI = 7.13–33.15), suggesting that IL-10 -1082A allele plays an important role in susceptibility to PEX, PEXG and POAG eye disorders. Furthermore, this finding shows a very little linkage disequilibrium between IL-10 -1082 position with -592C/A and -819C/T SNPs, suggesting that the effect is largely attributable to IL-10 -1082A/G polymorphism. Although, based on our findings it might be suggested that PEX, PEXG and POAG subjects having AA genotype of -592C/A, TT genotype of -819C/T and AA genotype of -1082A/G SNPs should be under control for possible Alzheimer’s disease, further studies on a greater number of patients and their long-term follow-up are required to confirm these results. Since both the Alzheimer’s disease and glaucoma are age-related, an increased understanding of the role of IL-10 in these three eye disorders may open the door to future treatments.

## Supplementary information

**Additional file 1: Table S1.** The primers sequence used and PCR products size. **Table S2.** PCR conditions used for amplifying the IL-10 gene SNPs

## Data Availability

The datasets generated and/or analysed during the current study are available in the figshare repository, [https://figshare.com/articles/Genotypic_distribution_of_three_polymorphisms_-592CA_-819CT_and_-1082AG_of_IL-10_gene_promoter_in_the_pathiets_with_PEX_PEXG_and_POAG_xlsx/11787243] (DOI: 10.6084/m9.figshare.11787243).

## Abbreviations

AMI: Acute myocardial infarction
ARMS-PCR: Amplification refractory mutation system-polymerase chain reaction
CI: Confidence interval
IBD: Inflammatory bowel disease
IL-10: Interleukin-10
IOP: Intraocular pressures
OR: Odds ratio
PEX: Pseudoexfoliation
PEXG: Pseudoexfoliative glaucoma
POAG: Primary open-angle glaucoma
SNPs: Single nucleotide polymorphisms

## References

[1] Ritch R, Schlötzer-Schrehardt U (2001). Exfoliation syndrome. Surv Ophthalmol.

[2] Konstas AGP, Stewart WC, Stroman GA, Sine CS (1997). Clinical presentation and initial treatment patterns in patients with exfoliation glaucoma versus primary open-angle glaucoma. Optalmic Surg Lasers Imaging Retina.

[3] Allingham RR, Loftsdottir M, Gottfredsdottir MS, Thorgeirsson E, Jonasson F, Sverisson T (2001). Pseudoexfoliation syndrome in Icelandic families. Brit J Ophthalmol.

[4] Aung T, Ozaki M, Lee MC, Schlötzer-Schrehardt U, Thorleifsson G, Mizoguchi T (2017). Genetic association study of exfoliation syndrome identifies a protective rare variant at LOXL1 and five new susceptibility loci. Nature Genet.

[5] Anastasopoulos E, Coleman AL, Wilson MR, Sinsheimer JS, Yu F, Katafigiotis S (2014). Association of LOXL1 polymorphisms with pseudoexfoliation, glaucoma, intraocular pressure, and systemic diseases in a Greek population. The Thessaloniki eye study. Invest Ophth Vis Sci.

[6] Gong WF, Chiang SWY, Chen LJ, Tam POS, Jia LY, Leung DYL (2008). Evaluation of LOXL1 polymorphisms in primary open-angle glaucoma in southern and northern Chinese. Mol Vis.

[7] Metaxaki I, Constantoulakis P, Papadimitropoulos M, Filiou E, Georgopoulos G, Chamchougia A (2013). Association of lysyl oxidase-like 1 gene common sequence variants in Greek patients with pseudoexfoliation syndrome and pseudoexfoliation glaucoma. Mol Vis.

[8] Chatzikyriakidou A, Founti P, Melidou A, Minti F, Bouras E, Anastasopoulos E (2018). MicroRNA-related polymorphisms in pseudoexfoliation syndrome, pseudoexfoliative glaucoma, and primary open-angle glaucoma. Ophthalmic Genet.

[9] Ovodenko B, Rostagno A, Neubert TA, Shetty V, Thomas S, Yang A (2007). Proteomic analysis of exfoliation deposits. Invest Ophthalmol Vis Sci.

[10] Wang R, Wiggs JL. Common and rare genetic risk factors for glaucoma. Cold Spring Harb Perspect Med. 2014;4:a017244. 10.1101/cshperspect.a017244. 10.1101/cshperspect.a017244 PMC4292091 25237143

[11] Zenkel M, Lewczuk P, Jünemann A, Kruse FE, GOH N, Schlötzer-Schrehardt U (2010). Proinflammatory cytokines are involved in the initiation of the abnormal matrix process in pseudoexfoliation syndrome/glaucoma. Am J Pathol.

[12] Farouk S, Salih MA, Musa AM, Blackwell JM, Miller EN, Khalil EA (2010). Interleukin 10 gene polymorphisms and development of post kala-azar dermal leishmaniasis in a selected sudanese population. Public Health Genomics.

[13] Turner DM, Williams DM, Sankaran D, Lazarus M, Sinnott PJ, Hutchinson IV (1997). An investigation of polymorphism in the interleukin-10 genepromoter. Eur J Immunogenet.

[14] Okayama N, Hamanaka Y, Suehiro Y, Hasui Y, Nakamura J, Hinoda Y (2005). Association of interleukin-10 promoter single nucleotide polymorphisms −819 T/C and −592 a/C with aging. J Gerontol.

[15] Khan MT, Sahar A, Rehman A, Zeb T (2015). Interleukin 10 (IL-10) promoter-1082 a>G polymorphism and risk of cancer: meta-analysis. Adv Life Sci.

[16] Li CX, Tong WD, Liu BH, Zhang AP, Li F. The -1082A.G polymorphism in promoter region of interleukin-10 and risk of digestive cancer: a meta-analysis. Sci Rep. 2014;4:5335. 10.1038/srep05335. 10.1038/srep05335 PMC4121615 25091209

[17] Liu L, Zheng F (2016). IL-10 -1082A/G, −592C/a, and -819T/C polymorphisms in association with lung cancer susceptibility: a meta-analysis. Onco Targets Ther.

[18] Gao L, Li Z, Chang S, Wang J. Association of interleukin-10 polymorphisms with schizophrenia: a meta-analysis. PLoS One. 2014;9(3):e90407. 10.1371/journal.pone.0090407. 10.1371/journal.pone.0090407 PMC3946087 24603720

[19] Arosio B, Trabattoni D, Galimberti L (2004). Interleukin-10 and interleukin-6 gene polymorphisms as risk factors for Alzheimer’s disease. Neurobiol Aging.

[20] Bagnoli S, Cellini E, Tedde A, Nacmias B, Piacentini S, Bessi V (2007). Association of IL10 promoter polymorphism in Italian Alzheimer's disease. Neurosci Lett.

[21] Magalhães CA, Carvalho MG, Sousa LP, Caramelli P, Gomes KB (2017). Alzheimer’s disease and cytokine IL-10 gene polymorphisms: is there an association?. Arq Neuropsiquiatr.

[22] Scassellati C, Zanardini R, Squitti R, Bocchio-Chiavetto L, Bonvicini C, Binetti G (2004). Promoter haplotypes of interleukin-10 gene and sporadic Alzheimer’s disease. Neurosci Lett.

[23] Zhang Y, Zhang J, Tian C, Xiao Y, Li X, He C (2011). The -1082G/a polymorphism in IL-10 gene is associated with risk of Alzheimer’s disease: a meta-analysis. J Neurol Sci.

[24] Chenjiao Y, Zili F, Haibin C, Ying L, Sheng X, Lihua H (2013). IL-10 promoter polymorphisms affect IL-10 production and associate with susceptibility to acute myeloid leukemia. Pharmazie.

[25] Mijac D, Petrovic IV, Djuranovic S, Perovic V, Bojic D, Culafic D (2016). The polymorphism rs3024505 (C/T) downstream of the IL10 gene is associated with Crohn’s disease in Serbian patients with inflammatory bowel disease. Tohoku J Exp Med.

[26] Zhang Y, Xing Y, Chen Z, Ma X, Lu Q (2019). Association between interleukin-10 genetic polymorphisms and risk of primary open angle glaucoma in a Chinese Han population: a case-control study. Int J Ophthalmol.

[27] Rosdahl JA, Muir KW (2015). Finding the best glaucoma questionnaire: a qualitative and quantitative evaluation of glaucoma knowledge assessments. Clin Ophthalmol.

[28] Anderson D. Automated static perimetry. St. Louis: MosbyYear Book (p 12), 1992.

[29] Hassanzad M, Farnia P, Ghanavi J, Parvini F, Seif S, Velayati AA (2018). TNFα −857 C/T and TNFR2 +587 T/G polymorphisms are associated with cystic fibrosis in Iranian patients. Eur J Med Genet.

[30] Abanmi A, Al Harthi F, Zouman A, Kudwah A, Jamal MA, Arfin M (2008). Association of Interleukin-10 gene promoter polymorphisms in Saudi patients with Vitiligo. Dis Markers.

[31] Aung T, Ozaki M, Mizoguchi T, Allingham RR, Li Z, Haripriya A (2015). A common variant mapping to CACNA1A is associated with susceptibility to exfoliation syndrome. Nature Genet.

[32] Gonzalez P, Epstein DL, Borra’s T (2000). Genes upregulated in the human trabecular meshwork in response to elevated intraocular pressure. Invest Ophthalmol Vis Sci.

[33] Paradowska-Gorycka A, Trefler J, Maciejewska-Stelmach J, Lacki JK (2010). Interleukin-10 gene promoter polymorphism in polish rheumatoid arthritis patients. Int J Immunogenet.

[34] Ying B, Shi Y, Pan X, Song X, Huang Z, Niu Q (2011). Association of polymorphisms in the human IL-10 and IL-18 genes with rheumatoid arthritis. Mol Biol Rep.

[35] Nie W, Fang Z, Li B, Xiu QY (2012). Interleukin-10 promoter polymorphisms and asthma risk: a meta-analysis. Cytokine.

[36] Cacev T, Radosevic S, Krizanac S, Kapitanovic S (2008). Influence of interleukin-8 and interleukin-10 on sporadic colon cancer development and progression. Carcinogenesis.

[37] Faupel-Badger JM, Kidd LC, Albanes D, Virtamo J, Woodson K, Tangrea JA (2008). Association of IL-10 polymorphisms with prostate cancer risk and grade of disease. Cancer Causes Control.

[38] Zhang G, Manaca MN, McNamara-Smith M, Mayor A, Nhabomba A, Berthoud TK (2012). Interleukin-10 (IL-10) polymorphisms are associated with IL-10 production and clinical malaria in young children. Infect Immun.

[39] Temple SE, Lim E, Cheong KY, Almeida CA, Price P, Ardlie KG (2003). Alleles carried at positions −819 and −592 of the IL10 promoter affect transcription following stimulation of peripheral blood cells with Streptococcus pneumoniae. Immunogenetics.

[40] Hamdan AA, Melconian AK, Adhia AH, Alhaidary AF (2014). The level of IL-1α, IL-10 and IL-17A in Alzheimer's disease patients: comparative study. Baghdad Sci J.

[41] Chakrabarty P, Li A, Ceballos-Diaz C, Eddy JA, Funk CC, Moore B (2015). IL-10 alters immunoproteostasis in APP mice, increasing plaque burden and worsening cognitive behavior. Neuron.

[42] Sestier MVG, Doty KR, Gate D, Rodriguez J, Leung BPY, Zadeh KR (2015). Il10 deficiency re-balances innate immunity to mitigate Alzheimer-like pathology. Neuron.

[43] Tsolaki F, Gogaki E, Tiganita S, Skatharoudi C, Lopatatzidi C, Topouzis F (2011). Alzheimer’s disease and primary open-angle glaucoma: is there a connection?. Clin Ophthalmol.

[44] Ghiso JA, Doudevski I, Ritch R, Rostagno AA (2013). Alzheimer’s disease and Glaucoma: mechanistic similarities and differences. J Glaucoma.

[45] McKinnon SJ (2003). Glaucoma:ocular alzheimer’s disease?. Front Biosci.

[46] Meretoja J, Tarkkanen A (1977). Occurrence of amyloid in eyes with pseudo-exfoliation. Ophthalmic Res.

