# Supplementary material for: Association of IL-10 gene promoter polymorphisms with susceptibility to pseudoexfoliation syndrome, pseudoexfoliative and primary open-angle glaucoma
Source: BMC Med Genet. 2020 Feb 12;21:32. doi: 10.1186/s12881-020-0969-6 (PMC7017446; doi:10.1186/s12881-020-0969-6)
Supplement: Supplementary file 1 — Additional file 1: Table S1. The primers sequence used and PCR products size. Table S2. PCR conditions used for amplifying the IL-10 gene SNPs [file 12881_2020_969_MOESM1_ESM.docx]

| **Table S1** The primers sequence used and PCR products size | | | |
| --- | --- | --- | --- |
| Gene | Polymorphism | Sequence (5’ 3’) | Amplicon size (bp) |
| IL-10 | -1082 A/G | F_N_: 5'- ACTACTAAGGCTTCTTTGGGAA -3' | 550 |
|  |  | F_M_: 5'- TACTAAGGCTTCTTTGGGAG -3' |  |
|  |  | R: 5'- CAGCCCTTCCATTTTACTTTC -3' |  |
|  | -592 C/A  -819 C/T | F_N_: 5'- CCCTTGTACAGGTGATGTAAC -3' | 233 |
|  |  | F_M_: 5'- ACCCTTGTACAGGTGATGTAAT -3' |  |
|  |  | R: 5'- AGGATGTGTTCCAGGCTCCT -3' |  |
| HGFH | - | F_C_: 5’- GCCTTCCCAACCATTCCCTTA -3’ | 429 |
|  |  | R_C_: 5’- TCACGGATTTCTGTTGTTTTC -3’ |  |

| **Table S2** PCR conditions used for amplifying the *IL-10* gene SNPs | | | | | |
| --- | --- | --- | --- | --- | --- |
| -592 C/A | | -819 C/T | | -1082 A/G | |
| 1 cycle | 94 ºC for 3 min | 1 cycle | 94 ºC for 4 min | 1 cycle | 94 ºC for 4 min |
| 35 cycles | 94 ºC for 40 sec  55 ºC for 40 sec  72 ºC for 60 sec | 35 cycles | 94 ºC for 40 sec  60 ºC for 40 sec  72 ºC for 30 sec | 35 cycles | 94 ºC for 40 sec  68 ºC for 40 sec  72 ºC for 40 sec |
| 1 cycle | 72 ºC for 6 min | 1 cycle | 72 ºC for 6 min | 1 cycle | 72 ºC for 6 min |
